# Clinical outcomes and toxicity of proton beam therapy for advanced cholangiocarcinoma

**DOI:** 10.1186/1748-717X-9-26

**Published:** 2014-01-14

**Authors:** Chiyoko Makita, Tatsuya Nakamura, Akinori Takada, Kanako Takayama, Motohisa Suzuki, Yojiro Ishikawa, Yusuke Azami, Takahiro Kato, Iwao Tsukiyama, Yasuhiro Kikuchi, Masato Hareyama, Masao Murakami, Nobukazu Fuwa, Masaharu Hata, Tomio Inoue

**Affiliations:** 1 Department of Radiation Oncology, Southern Tohoku Proton Therapy Center, 7-172, Yatsuyamada, 963-8052 Koriyama, Fukushima, Japan; 2 Department of Radiology, Graduate School of Medicine, Dokkyo Medical University, 880 Kitakobayashi, Mibu-machi, 321-0293 Shimotsuga, Tochigi, Japan; 3 Hyogo ion beam Medical Center, 1-2-1 Koto, Shingu, 679-5165 Tatsuno, Hyogo, Japan; 4 Department of Radiology, Graduate School of Medicine, Yokohama City University, 3-9 Fukuura, 236-0004 Kanazawa-ku, Yokohama, Japan

**Keywords:** Cholangiocarcinoma, Proton beam therapy, Chemoradiotherapy, Cholangitis, Gastrointestinal toxicity

## Abstract

**Background:**

We examined the efficacy and toxicity of proton beam therapy (PBT) for treating advanced cholangiocarcinoma.

**Methods:**

The clinical data and outcomes of 28 cholangiocarcinoma patients treated with PBT between January 2009 and August 2011 were retrospectively examined. The Kaplan–Meier method was used to estimate overall survival (OS), progression-free survival (PFS), and local control (LC) rates, and the log-rank test to analyze the effects of different clinical and treatment variables on survival. Acute and late toxicities were assessed using the National Cancer Institute Common Terminology Criteria for Adverse Events version 4.0.

**Results:**

The median age of the 17 male and 11 female patients was 71 years (range, 41 to 84 years; intrahepatic/peripheral cholangiocarcinoma, n = 6; hilar cholangiocarcinoma/Klatskin tumor, n = 6; distal extrahepatic cholangiocarcinoma, n = 3; gallbladder cancer, n = 3; local or lymph node recurrence, n = 10; size, 20–175 mm; median 52 mm). The median radiation dose was 68.2 Gy (relative biological effectiveness [RBE]) (range, 50.6 to 80 Gy (RBE)), with delivery of fractions of 2.0 to 3.2 Gy (RBE) daily. The median follow-up duration was 12 months (range, 3 to 29 months). Fifteen patients underwent chemotherapy and 8 patients, palliative biliary stent placement prior to PBT. OS, PFS, and LC rates at 1 year were 49.0%, 29.5%, and 67.7%, respectively. LC was achieved in 6 patients, and was better in patients administered a biologically equivalent dose of 10 (BED10) > 70 Gy compared to those administered < 70 Gy (83.1% vs. 22.2%, respectively, at 1 year). The variables of tumor size and performance status were associated with survival. Late gastrointestinal toxicities grade 2 or greater were observed in 7 patients <12 months after PBT. Cholangitis was observed in 11 patients and 3 patients required stent replacement.

**Conclusions:**

Relatively high LC rates after PBT for advanced cholangiocarcinoma can be achieved by delivery of a BED10 > 70 Gy. Gastrointestinal toxicities, especially those of the duodenum, are dose-limiting toxicities associated with PBT, and early metastatic progression remains a treatment obstacle.

## Background

Cholangiocarcinoma is a malignant tumor arising from the epithelium of the bile ducts. It is estimated that 23,000 cases of cholangiocarcinoma are diagnosed in Japan every year, an incidence higher than that in western countries [1]. Although surgery is the only potentially curative treatment for cholangiocarcinoma, only 10% to 30% patients are candidates for surgery at presentation [2]. The majority of patients present with either locally advanced or distant metastatic disease. Even for patients in whom curative resection is performed, the prognosis remains poor; local failure rates are high, and death from biliary obstruction, sepsis, and liver failure is common. For patients with unresectable cholangiocarcinoma, survival is poor, ranging from 3 to 9 months in those receiving medical management alone due to tumor aggressiveness [2-4].

Treatment of cholangiocarcinoma by conventional of radiotherapy is controversial for several reasons [5]. These include frequent local and regional recurrence despite provision of definitive chemoradiotherapy; radiation intolerance, which limits the dose that may be delivered to the entire liver, proximal biliary tract, and digestive tract [6]; and inability to deliver high dose X-rays to the tumor. However, using charged-particle therapy, superior dose distribution can be achieved due to the existence of the Bragg peak, which enables delivery of higher doses of radiation to tumor tissue without increasing exposure to the surrounding normal tissue. Schoenthaler et al. reported providing charged-particle therapy using helium and/or neon to treat 22 patients with extrahepatic bile duct carcinoma [7]. Improved median survival has been observed in patients with microscopic residual disease with the addition of adjuvant irradiation after charged-particle therapy, also in comparison to patients treated with conventional radiotherapy. Since January 2009, advanced cholangiocarcinoma has been treated at our institution with proton beam therapy (PBT), a form of charge-particle therapy. Despite the potential of this form of treatment, to our knowledge no other study has examined its use in treating this patient population. To fill this research gap, this study retrospectively evaluated the efficacy and toxicity of PBT for the treatment of unresectable and inoperable cholangiocarcinoma.

## Methods

### Patients

Between January 2009 and August 2011, 28 patients with advanced cholangiocarcinoma (18 patients with unresectable cholangiocarcinoma and 10 with recurrent tumor after surgery) were treated with PBT at our institution. The initial workup for these patients had generally included taking a medical history; performing a physical examination; conducting laboratory testing for a comprehensive metabolic panel and measurement of complete blood cell count, carcinoembryonic level, and carbohydrate antigen 19–9 level; and conducting chest X-ray, electrocardiogram, computed tomography (CT) with positron emission tomography (PET) using 2-(fluorine-18)-fluoro-2-deoxy-D-glucose (FDG-PET/CT), enhanced abdominal CT. Magnetic resonance imaging (MRI) was performed on some patients to get additional information. Histological diagnosis of cholangiocarcinoma had been determined in all newly diagnosed patients prior to initiation of PBT. Pathological diagnosis of recurrent cases had been made prior to surgery.

Biopsy procedures had typically involved endoscopic retrograde cholangiopancreatography with or without endoscopic ultrasonography. Resectability had been determined by individual hepatic surgeons. No evidence of distant metastases had been found at the time of PBT initiation in any patient. Abdominal surgical staging had been performed in 1 patient, biliary stenting to relieve symptoms in 8 patients with biliary obstruction, ultrasound-guided percutaneous transhepatic biliary drainage in 3 patients, and endoscopic retrograde biliary drainage in 5 patients. Patients with carcinoma of the ampulla of Vater were excluded from this study.

### Treatment

All patients had undergone simulation using a 16-slice large-bore helical CT scanner (Aquilion LB; Toshiba, Tokyo, Japan) and a respiratory gating system (Anzai Medical, Tokyo, Japan). Using this system, CT images had been obtained in the exhalation phase, and a conventional scan with a 2-mm slice thickness had been obtained. A custom-induced vacuum-lock bag (Esform; Engineering System Co., Matsumoto, Japan) had been used for patient immobilization. Diagnostic CT or MRI images had been fused with planning CT images for target delineation. For PBT planning, a 3-dimensional treatment planning system (Xio-M; CMS Japan, Tokyo, Japan; Mitsubishi Electric Corporation, Kobe, Japan) had been used. Gross tumor volume had been identified from review of these images by the liver surgeon, gastroenterologist, and diagnostic radiologists.

The clinical target volume had included a 5- to 10-mm radial expansion of the gross tumor volume to target possible microscopic disease extension. Regional lymph nodes had not been intentionally covered unless pathologically enlarged. The planning target volume had been expanded by 5 mm in all directions to create an additional 5- to 7-mm margin in the craniocaudal direction to compensate for respiratory movements. The photon plan was compared with the dose–volume histogram, although conventional planning target volume is not typically used in proton planning. The total dose at the isocenter had been prescribed to cover 90% of the planning target volume. Doses had been calculated on the basis of the pencil beam algorithm. Proton energy levels of 150 and 210 MeV for 1 to 4 portals had been planned.

The PBT system (Proton Therapy System; Mitsubishi) uses a synchrotron that could accelerate protons up to 235 MeV. A respiratory gating system (Anzai Medical, Tokyo, Japan) had been used to synchronize treatment in the expiratory phase. The relative biological effectiveness of the proton beam had been determined to be 1.1. The median radiation dose had been 68.2 Gy (relative biological effectiveness [RBE]) (range, 50.6 to 80 Gy (RBE)), with delivery of fractions of 2.0 to 3.2 Gy (RBE) daily, depending on tumor location. In cases in which the tumor had been adjacent to the digestive tract, a dose of 2.0 to 2.2 Gy (RBE) per fraction had been delivered. In cases in which the tumor had been greater than 2 cm from the gastrointestinal tract and porta hepatis, a dose of 3.2 Gy (RBE) per fraction had been delivered.

### Follow-up and toxicity evaluation

Abdominal imaging studies (CT, MRI, or FDG-PET/CT) and lab analyses (including measurement of tumor marker levels) had been performed every 3 months after PBT for the first 2 years. In cases in which obstructive jaundice and hyperbilirubinemia had persisted after completion of PBT, additional radiological analyses had been performed to evaluate local failure. In the subsequent analysis of outcomes, local control (LC) was defined as no sign of regrowth or new tumor development in the target area. Acute and late toxicities were assessed using the National Cancer Institute Common Terminology Criteria for Adverse Events version 4.0. To evaluate post-treatment late toxicity in the duodenum, dose–volume histograms were developed for cases delivered a BED of 3.

### Statistical analysis

Observation of endpoints began on the same date on which proton therapy was initiated. The Kaplan–Meier method was used to estimate overall survival (OS), progression-free survival (PFS), and LC and the log-rank test to analyze the effects of different clinical and treatment variables on survival. All statistical analyses were performed using SPSS software version 18.0 (SPSS, Chicago, IL, USA).

### Ethical approval

This study was approved by an institutional committee of Southern Tohoku Proton Therapy Center. The research was in compliance with the Helsinki Declaration.

## Results and discussion

### Patient, tumor, and treatment characteristics

Table 1 lists the patient and tumor characteristics. The median age of the 17 male and 11 female patients had been 71 years (range, 41 to 84 years). Of the 28 patients, 6 had been newly diagnosed with intrahepatic cholangiocarcinoma, 6 with hilar cholangiocarcinoma (Klatskin tumor), 3 with distal extrahepatic cholangiocarcinoma, and 3 with gallbladder cancer. Seven patients had experienced local recurrence and 3 lymph node recurrence. PBT had been administered to recurrent cases 12 to 40 months after surgery. Tumors treated with PBT had ranged in size from 20 to 175 mm (median, 52 mm) in the greatest dimension.

**Table 1:** Patient and tumor characteristics

	**N or range**	**% or median**
No. of patients	28	100
Age (years)	48–89	78
Sex		
Male	17	61
Female	11	39
Tumor size, maximum diameter (mm)	20–175	52
ECOG* performance status		
0	15	54
1	8	29
2	5	18
Tumor location		
Intrahepatic	6	21
Extrahepatic	-	-
Hilar	6	21
Distal	3	11
Gall bladder	3	11
Tumor recurrence after surgery		
Local	7	25
Nodal	3	11
CA19-9†		
<37	13	46
≥37	15	54
Palliative stent placement		
No	20	71
Yes	8	29

*Eastern Cooperative Oncology Group; †carbohydrate antigen 19–9 level.

Table 2 lists the treatment characteristics of the patients examined in this study. Because various fractionation regimens had been used for treatment, a biologically equivalent dose (BED) was calculated for comparison using the linear–quadratic model. Assuming a BED of 10 (i.e., BED10; α/β = 10) for tumor control, the estimated median BED10 had been 75.8 Gy (range, 61.7 to 105.6 Gy). Assuming a BED of 3 (BED3; α/β = 3) for cases in which late toxicity had been observed, the estimated median BED3 had been 106.8 Gy (range, 95.3 to 165.3 Gy). Chemotherapy consisting of gemcitabine and/or tegafur, gimeracil, and either oteracil (S-1) or cisplatin before or after PBT had been administered to 15 patients, and S-1 had been administered concurrently with PBT treatment to 3 patients.

**Table 2:** Patient treatment characteristics

	**N**	**%**
Total dose (Gy (RBE))		
50.6–60	7	43
61.6–70	11	21
70.4–80	10	11
Dose/fraction (Gy (RBE))		
2.0	2	7
2.2	20	71
2.4	5	18
3.2	1	4
BED10 (α/β = 10) (Gy)		
61.7–69.5	6	21
72.5–75.1	10	36
83.2–89.3	10	36
91.3–105.6	2	7
BED3 (α/β = 3) (Gy)		
87.7–98.8	6	21
103.0–108.0	10	36
116.7–118.2	3	11
122.2–165.3	9	32
History of chemotherapy		
None	18	64
Completed	10	36
Follow-up chemotherapy		
None	13	50
Concurrent	3	11
Adjuvant	15	50
Regimen		
S-1	5	18
GEM	7	25
S-1 + GEM	2	7
GEM + CDDP	1	4

BED, biologically effective dose; S-1, tegafur, gimeracil, and oteracil; CDDP, cisplatin; RBE, relative biological effectiveness.

### Clinical outcomes

**Figure 1:**
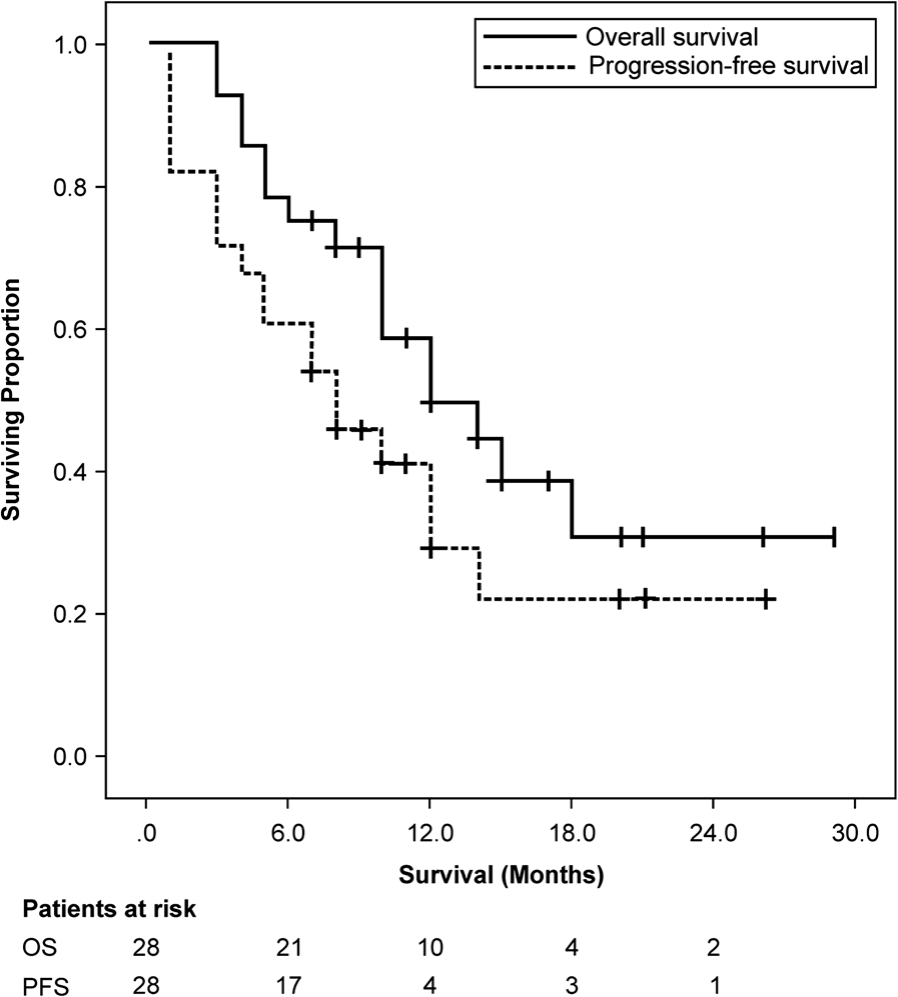
**Overall survival and progression-free survival.** Median overall survival was 12.0 months and median progression-free survival 8.0 months.

**Figure 2:**
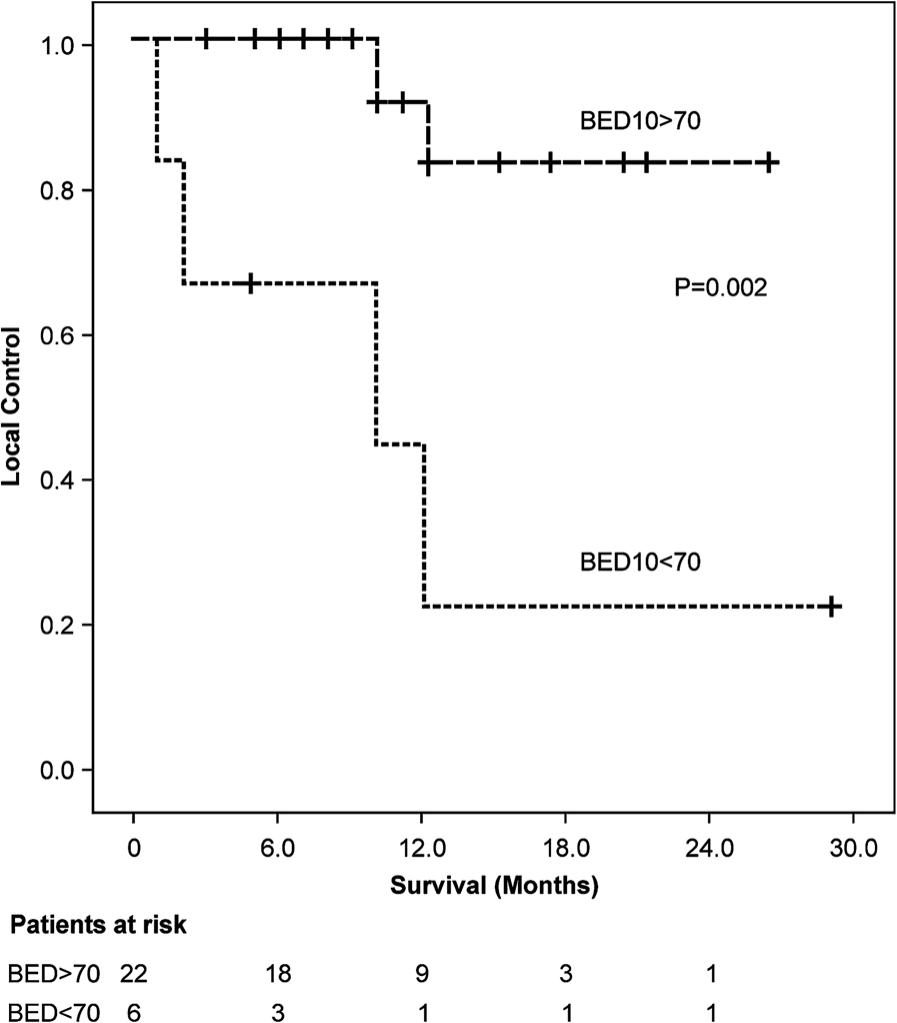
Local control stratified according administration of BED10 >70 Gy or < 70 Gy.

Among all patients, the median follow-up duration had been 12 months (range, 3 to 29 months) and OS and PFS at 1 year had been 49.0% (95% confidence interval [CI] 59.1% to 38.9%) and 29.5% (95% CI 39.3% to 19.7%), respectively (Figure 1). Univariate analysis identified a significant relationship between survival and the variables of tumor size and performance status**,** but not between survival and the variables of age, gender, tumor markers, and chemotherapy (Table 3). Of the 19 patients (67.9%) who had experienced recurrence during the observation period, 6 patients (21.4%) had developed local recurrence, 5 patients (17.9%) new intrahepatic tumors, 5 patients (17.9%) peritonitis carcinomatosa, 2 patients (7.1%) distant metastases, and 1 patient (3.6%) lymph node metastases. Of the 16 patients (57.1%) who had died during the observation period, the death of 14 patients (had been attributed to cholangiocarcinoma; the death of 1 patient (3.6%) to cholangitis-related disseminated intravascular coagulation after replacement of the biliary stent, which had caused perforation of the bile duct; and the death of 1 patient (3.6%) to lung cancer. The LC rate at 1 year had been 67.7% (95% CI 79.1 to 56.3). Univariate analysis indicated better LC in patients to whom a BED10 greater than 70 Gy PBT had been administered compared with those to whom a BED10 less than 70 Gy had been administered (83.1% vs. 22.2%, respectively, at 1 year; *p* = 0.002) (Figure 2).

**Table 3:** Univariate analysis of factors potentially affecting overall survival

	**n**	**Median survival (months)**	**p**
Age (years)			
≥70	14	10.0	0.034
<70	14	14.0	-
Sex			
Male	17	12.0	0.339
Female	11	14.0	-
ECOG performance status			
1 or 2	13	5	0.000
0	15	-	-
Tumor size (mm)			
>50	15	10.0	0.008
≤50	13	18.0	-
BED-10 (Gy)			
>70	22	14.0	0.243
<70	6	5.0	-
CA19-9			
>37	15	10.0	0.312
≤37	13	15.0	-
Chemotherapy			
Yes	15	18.0	0.474
No	13	12.0	-

### Toxicity

Table 4 lists the acute and late toxicities that had been experienced by the patients. Eight patients had developed acute treatment-related toxicities of grade 2 or greater in the forms of thrombocytopenia, erythema, anorexia, abdominal pain, diarrhea, and cholangitis. Six patients had developed late toxicities of cholangitis of grade 2 or 3. One patient had been diagnosed with grade 2 common bile duct stenosis. All patients with acute and late treatment-related cholangitis had been treated with intravenous antibiotics, and 3 had required stent replacement. Late gastrointestinal toxicities of grade 2 or greater had been experienced by 7 patients within 12 months after PBT in the forms of duodenal ulcer in 2 patients, duodenal hemorrhage in 2 patients, duodenal stenosis in 2 patients, and gastric ulcer in 1 patient. Of the 15 patients (53.6%) who had undergone irradiation of the duodenum, the mean irradiated volume exceeding a BED3 of 80 Gy to the duodenum had been 11.0 ± 4.3 ml. Of the 6 patients who had experienced duodenal-related toxicities, the mean irradiated volume exceeding a BED3 of 80 Gy to the duodenum had been 21.0 ± 5.1 ml.

**Table 4:** Acute and late toxicities experienced during observation period

	**Grade**				
**Toxicity**	**1**	**2**	**3**	**4**	**5**
Acute					
Leucopenia	5	0	0	0	0
Anemia	2	0	0	0	0
Thrombocytopenia	1	1	0	0	0
Erythema	7	1	0	0	0
Nausea	3	0	0	0	0
Anorexia	7	2	0	0	0
Abdominal pain	3	1	0	0	0
Diarrhea	0	1	0	0	0
Cholangitis	1	3	1	0	0
Late					
Cholangitis	0	4	2	0	0
Common bile duct stenosis	0	0	1	0	0
Gastric ulcer	0	1	0	0	0
Duodenal ulcer	0	1	1	0	0
Duodenal hemorrhage	0	0	2	0	0
Duodenal stenosis	0	1	1	0	0

In patients with unresectable cholangiocarcinoma, palliative irradiation following biliary decompression has been shown to prolong survival [3]. Although patients with postoperative local or regional recurrence are treated with radiotherapy, no definitive treatment has yet been established. Because no global consensus exists for treatment of advanced cholangiocarcinoma, various palliative therapies have been administered to attempt to improve survival and quality of life, including external beam radiotherapy (EBRT), brachytherapy, systemic chemotherapy, radiofrequency ablation, and transarterial chemoembolization [8,9]. Because of their ability to deliver markedly higher doses of radiation directly to tumor tissue, intensity-modulated radiotherapy and stereotactic body radiotherapy have also been utilized [10,11].

Among the treatment options that have been examined, one study investigated therapy using charged particles, such as neon and helium ions, in the treatment of extrahepatic bile duct carcinoma with microscopically positive margins after surgery in 22 patients [7]. Delivering a total dose ranging from 4800 to 6778 cGy (median, 6000 cGy) with curative intent, a trend toward improved outcome in patients treated with neon was observed, with the median survival of these patients found to be 25 months compared to 12.5 months with helium and 11 months with photon therapy.

In an effort to maximize outcomes in patients with advanced cholangiocarcinoma who are treated radiotherapeutically, several studies have investigated the presumed correlation between tumor dose and survival. Alden and Mohiduddin reported an improvement in the 2-year survival rate (48% vs. 0%, *p* = 0.03) and median survival duration (24 months vs. 6 months) with treatment with EBRT and brachytherapy delivering total doses greater than 55 Gy [12]. Valek et al. found that administration of intraluminal brachytherapy significantly prolonged survival and symptom-free duration in patients with unresectable cholangiocarcinoma who had undergone stent replacement [13]. Improved LC and OS have also been observed with higher radiation doses, suggesting that dose escalation may be a promising therapeutic approach. On the other hand, Crane et al. found that the addition of brachytherapy provided no benefit [14]. Although we did observe a better LC in patients administered a BED10 of greater than 70 Gy PBT compared to those receiving a BED10 of less than 70 Gy (89% vs. 36%, respectively, at 1 year), no survival benefit was found with administration of a higher proton dose in the current study.

The role of chemotherapy in treating cholangiocarcinoma is unclear. Kopleson et al. asserted the feasibility and potential benefit of chemotherapy in addition to radiation [15]. Deodato et al. also reported that concurrent chemotherapy with 5-fluorouracil appeared to improve outcome based on observation of a median survival of 22 months and a 2-year survival rate of 41% [16]. In contrast, Crane et al. found no survival benefit of concurrent chemoradiation [14]. In accordance with the findings of Crane et al. [14], combined chemotherapy and PBT was found to provide no survival benefits to the patients in the current study, in whom early metastatic progression of the liver, regional lymph nodes, and distant lesions remained major treatment obstacles. Among all patients in this study, 19 (67.9%) experienced tumor recurrence, with 13 (46.4%) of these 19 patients developing recurrent tumors outside of the proton-irradiated field, among them the 7 patients who had been undergone chemotherapy. Further study is needed to define the roles of chemotherapy and PBT in cholangiocarcinoma.

Potential acute toxicities of combined EBRT and chemotherapy include nausea, vomiting, anorexia, dehydration, skin reaction, gastritis, duodenitis, fatigue, and liver dysfunction [15-17]. In a study of a series of 81 patients with extrahepatic cholangiocarcinoma who underwent combined chemotherapy and radiotherapy, Ben-David et al. observed development of late complications (ulcer formation, gastritis, and/or veno-occlusive disease of the liver) in 5 patients with hilar carcinoma at a median of 6 months after treatment [17]. Regarding the effect of volume on the development of gastrointestinal toxicity, Emani et al. estimated a dose of 1/3 for small bowel irradiation (i.e., 50 Gy) based on the probability of a 5% complication within the 5 years following irradiation (i.e., a tolerance dose [TD] of 5/5). Emani et al.’s estimation remains a commonly applied dose limit when small portions of the small bowel are treated with a conventional fraction. Despite this estimation, the probability of a 50% complication within the 5 years following irradiation (TD50/5) for partial small bowel irradiation (i.e., delivery of 60 Gy) remains unexplored [18]. The minimum tolerable radiation dose to the duodenum that results in TD 5/5 is 50 Gy with fractions of 1.8 Gy/day, a dose equivalent to a BED3 of 80 Gy.

Although duodenal complications were observed in 6 patients in the current study, 15 of the 28 patients (53.6%) had undergone duodenal irradiation at a mean volume of BED3 of >80 Gy of 11.0 ± 4.3 ml. In the 6 patients in whom duodenal toxicities occurred, the irradiated volume to the duodenum (BED3 of >80 Gy) was 21.0 ± 5.1 ml. To minimize toxicity in patients with cholangiocarcinoma adjacent to the gastrointestinal tract, especially the duodenum, the irradiated volume of duodenal irradiation should be decreased in PBT.

Due to limitations of the present study such as small number of patients, heterogeneous doses, and retrospective nature, optimal PBT doses and chemotherapy regimen are still unclear. But we indicate the potential benefit and toxicity profile of PBT for advanced cholangiocarcinoma. Further investigations are warranted.

## Conclusion

In conclusion, the study results indicate that PBT is an effective, as evidenced by the high LC rates of patients administered a BED10 greater than 70 Gy, as well as feasible, as evidenced by the tolerable toxicity levels of all patients except those 6 who experienced duodenal toxicity, form of treatment for cholangiocarcinoma. Nevertheless, early metastatic progression remains a major obstacle since PBT may not be effective in those cases. Further study is needed to define the roles of chemotherapy and PBT in cholangiocarcinoma treatment.

## Abbreviations

BED: Biologically equivalent dose; LC: Local control; OS: Overall survival; PBT: Proton beam therapy; PFS: Progression-free survival; RBE: Relative biological effectiveness.

## Competing interest

The authors declare that they have no competing interests.

## Authors’ contributions

TN, AT, KT, MS, YI, and YA treated all of the patients with PBT. NF participated in the design of the study. TK checked and calculated all the plans. MM, IT, MM and MH conceived the study, participated in its design and coordination, and helped to draft the manuscript. All authors read and approved the final manuscript.
